# Phytochemical Composition and Mechanistic Pharmacology of Jerusalem Artichoke (*Helianthus tuberosus* L.): Implications for Functional and Therapeutic Applications

**DOI:** 10.3390/cimb48020180

**Published:** 2026-02-05

**Authors:** Dong-Hwan Kim, Wonmin Lee, Yeonhee Pyo, Dong-Kug Choi

**Affiliations:** 1 Department of Beauty Fragrance, Graduate School of Bio-Wellness Convergence, Konkuk University, Chungju 27478, Republic of Korea; 2 Scentcos Inc., Institute of Cosmetics, Pyeongtaek-si 17911, Republic of Korea; 3 Department of Biotechnology, College of Biomedical and Health Science, Konkuk University, Chungju 27478, Republic of Korea

**Keywords:** Jerusalem artichoke (JA), bioactive compounds, pharmacology, antioxidant activity, anti-inflammatory

## Abstract

Jerusalem artichoke (JA) (*Helianthus tuberosus*), a perennial plant of the *Asteraceae* family, is well known for its high inulin content and diverse bioactive compounds, including flavonoids, phenolic acids, sesquiterpenes, and amino acids. Extracts derived from different parts of JA, such as tubers, leaves, and flowers, have demonstrated a wide range of biological activities, including antioxidant, anti-inflammatory, antihyperglycemic, antihypertensive, and antifungal effects. These properties highlight JA’s potential in the prevention and management of chronic diseases such as diabetes, cardiovascular disorders, obesity, and colorectal cancer. Recent studies also suggest that JA benefits skin health through anti-aging and barrier-protective mechanisms and enhances immune function by modulating the intestinal microbiota. Owing to its multifunctional physiological activities, JA is being explored as a valuable raw material for food, nutraceutical, cosmetic, and pharmaceutical applications. However, most existing research has focused primarily on inulin, while comprehensive studies on other bioactive constituents and their clinical validation remain limited. This paper aims to provide a comprehensive overview of the bioactive compounds present in JA, elucidate their health-promoting functions, discuss their pharmacokinetics, and outline future perspectives on their potential as functional ingredients and biohealth materials.

## 1. Introduction

Natural products continue to play a crucial role in human health, modern medicine, and drug discovery. According to the World Health Organization (WHO), nearly 80% of the global population relies on natural medicines, and many modern drugs originate from medicinal plants. The search for bioactive molecules from natural sources has led to the discovery of key therapeutic agents with antioxidant, anti-inflammatory, analgesic, antibiotic, and anticancer properties [1]. The vast genetic diversity of medicinal plants offers valuable opportunities to develop nutritionally and pharmacologically active ingredients. Currently, the global phytotherapy and pharmacology market is valued at about $14 billion annually, representing roughly 5% of the $280 billion traditional medicine sector. Notably, plant-based products account for 80% of medication use in developing countries, compared to 25% in developed nations [2]. Of all synthetic drugs, about 56% are linked to natural sources—24% derived from plants, 9% synthesized using natural templates, and 6% directly isolated from natural species. Despite this progress, vast potential remains unexplored, as only about 20% of the estimated 350,000–550,000 plant species have been investigated for therapeutic use [3,4].

Jerusalem artichoke (JA) (*Helianthus tuberosus* L.) is a perennial herbaceous plant belonging to the Asteraceae family. It is widely cultivated in temperate regions worldwide, especially in the northeastern, northwestern, and northern provinces of China [5]. JA contains inulin and various phenolic compounds, and as a result, its functionality related to improved metabolic health, anti-inflammatory effects, and antioxidant effects has been reported [6,7,8,9,10,11]. Interestingly, consuming JA in the morning has a stronger blood glucose-lowering effect than evening intake, leading to a marked reduction in blood sugar levels. Beyond its metabolic benefits, JA exhibits antioxidant, anti-inflammatory, antihypertensive, and antifungal activities, helping prevent chronic diseases such as diabetes, cardiovascular disease, and obesity. JA hydrolysate has demonstrated antidiabetic activity by reducing low-grade oligosaccharides, increasing reducing sugars, and enhancing enzyme activity [12]. Additionally, JA has been reported to possess functional potential related to skin health and mineral balance [13]. Despite its benefits, important gaps still exist in fully understanding its therapeutic potential, pharmacological mechanisms, and phytochemistry.

Given the increasing interest in the health benefits of JA, this paper presents a systematic and comprehensive review of its pharmacological properties and the health effects of its bioactive compounds. It also highlights JA’s diverse applications in the food industry, including its incorporation into dairy products, snacks, baked goods, beverages, and functional foods. In addition, this review discusses current research gaps and proposes directions for future studies to promote the broader academic and industrial utilization of JA.

Previous reviews on JA have primarily focused on its nutritional value centered on inulin, while integrated interpretations of the mechanisms, pharmacological actions, and functional applications of individual bioactive components have been relatively limited. As a result, gaps remain in our comprehensive understanding of the pharmacological significance and potential applications of the individual bioactive components contained in JA. To address these limitations, this study systematically reviews research published from 2020 to 2025 according to the PRISMA 2020 guidelines and comprehensively analyzes the phytochemical composition of JA and its experimentally validated pharmacological mechanisms of action. Specifically, by operationalizing the scope of research based on the type of bioactive components, biological targets, and application fields, we aim to organically integrate nutritional and pharmacological perspectives—unlike existing reviews.

This review aims to synthesize the latest evidence on the functionality and therapeutic potential of JA through this systematic approach, and to present directions for future translational research and industrial applications by comparing and analyzing the consistency and differences among research findings.

A comprehensive literature search was performed using major web-based electronic databases, including PubMed, Web of Science, Embase, SCOPUS, and Google Scholar. The search focused on peer-reviewed literature published between January 2020 and December 2024. The final search was completed on 4 April 2024, and all eligible references were recorded with their corresponding DOIs.

The search strategy employed a combination of controlled vocabulary and free-text terms appearing in the title or abstract. The primary search keywords included “Jerusalem artichoke” OR “*Helianthus tuberosus*” AND “phytochemistry” OR “bioactive compounds” OR “inulin” OR “polyphenols” OR “flavonoids” AND “pharmacological activity” OR “anti-inflammatory” OR “antioxidant” OR “metabolic health” OR “functional applications”. Boolean operators “AND” and “OR” were applied as appropriate.

Studies were included if they met the following criteria: Original experimental or clinical studies investigating *Helianthus tuberosus* or its bioactive constituents. In vitro, in vivo (animal), or human studies reporting phytochemical composition, pharmacological activity, or functional health effects. Articles written in English and published within the defined time frame (2014–2024).

Studies were excluded if they were review articles, case reports, theses, conference abstracts, editorials, or letters; were not available in full text; focused solely on agronomic or botanical traits without biochemical or functional relevance; or presented clinically ambiguous or methodologically insufficient data.

The study selection process was conducted independently by two reviewers, who screened titles and abstracts of all retrieved records for relevance. Full-text articles were subsequently assessed against the inclusion and exclusion criteria. A standardized data extraction form was used to collect key information, including study design, bioactive compounds, experimental models, mechanisms of action, and reported functional or therapeutic outcomes. Any discrepancies between reviewers were resolved through consultation with a third reviewer.

The methodological quality of the included studies was assessed using the Cochrane Collaboration risk of bias tool for randomized studies and the Newcastle–Ottawa Scale (NOS) for non-randomized studies. This evaluation was conducted to review the reliability of the included studies’ findings and critically identify methodological factors that could influence differences in results across studies. The assessment criteria included study design, sample size, data collection methods, clarity of outcome reporting, and appropriateness of statistical analyses.

Risk of bias was evaluated across multiple domains, including randomization procedures, allocation concealment, blinding of participants and outcome assessors, completeness of outcome data, selective reporting, and other potential sources of bias. Each domain was rated as low, high, or unclear risk.

**Figure 1:**
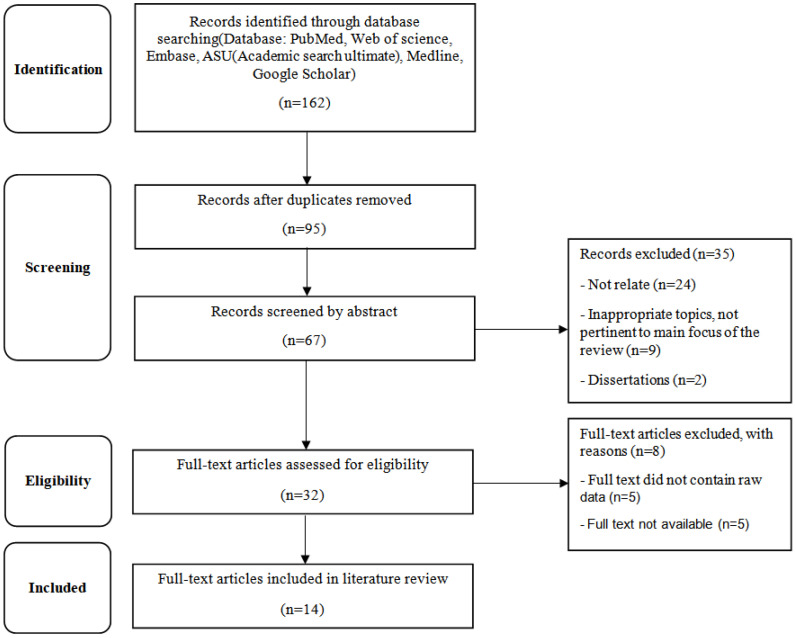
PRISMA.

A total of 162 records were initially identified from PubMed (*n* = 34), Web of Science (*n* = 28), Embase (*n* = 19), SCOPUS (*n* = 41), and Google Scholar (*n* = 40). After removing duplicates (*n* = 67), 95 articles were screened based on titles and abstracts. Following full-text assessment, 81 studies were excluded, and 14 studies met the inclusion criteria and were included in the final qualitative synthesis. The study selection process is illustrated in the PRISMA 2020 flow diagram Figure 1.

The risk of bias assessment revealed recurring methodological limitations across all included studies. For randomized and clinical trials assessed using the Cochrane tool, numerous instances of inadequate reporting were observed regarding the randomization procedure, allocation concealment, and blinding of participants and outcome assessors. Consequently, these studies were classified as having an ‘uncertain risk’ of bias across multiple assessment domains.

Studies assessed using the Newcastle–Ottawa Scale consistently showed relatively low scores in the areas of comparability of treatment groups and control of confounding variables. These findings suggest that despite many studies providing functional or mechanistic evidence, they contain certain limitations in terms of the rigor of their study design and reporting.

The risk of bias identified in this review significantly impacts the interpretation of results regarding JA’s physiological activity and therapeutic potential. In particular, the absence of random assignment, blinding, and standardized outcome measures makes it difficult to rule out the possibility that the effects reported in some studies may have been overestimated. Furthermore, when studies examining similar bioactive substances reported divergent outcomes, differences in experimental design and analytical methods were observed alongside varying levels of risk of bias. This methodological heterogeneity is considered a factor that may partially explain inconsistencies between study results.

Future studies should systematically incorporate rigorous research designs, including randomization and blinding, standardized extracts and dosage settings, and sufficient sample sizes to enhance reproducibility and reliability.

## 2. Taxonomy, Distribution and Description of JA

**Figure 2:**
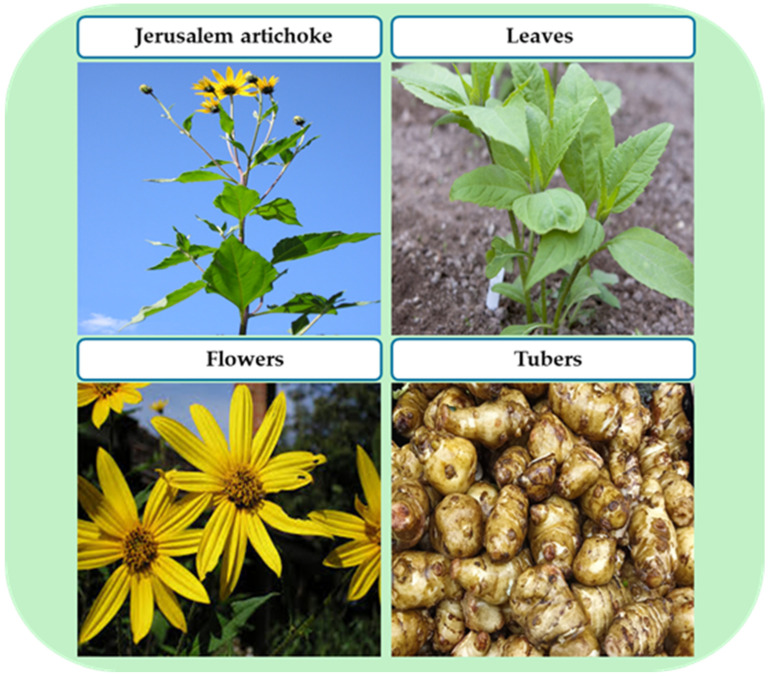
Schematic representation of the health benefits of Jerusalem artichoke (JA) from different parts of consumption.

The genus *Helianthus*, belonging to the family Asteraceae and the subfamily Asteroideae [14], comprises approximately 50 species, of which only two are widely cultivated for their medicinal and nutritional value: Jerusalem artichoke (JA) and *Helianthus annuus* [15]. *H. annuus* is primarily grown as an oilseed crop valued for its nutritional properties [16], whereas JA is a perennial tuberous plant cultivated globally for its diverse beneficial traits [17]. JA thrives in various soil types without the need for chemical fertilizers or pesticides. It exhibits strong frost resistance, requires minimal maintenance, and shows high tolerance to pests and diseases, features that have attracted significant scientific interest [18]. The plant grows well in semi-arid tropical regions [19], with optimal growth occurring under annual precipitation levels of 31–282 cm and temperatures ranging from 6.3 to 26.6 °C (43.3–79.9 °F) at soil pH values between 4.5 and 8.2 [20]. These differences in cultivation environments have been reported to affect not only the growth characteristics of Jerusalem artichoke but also the content and composition of key bioactive compounds, such as inulin, phenolic compounds, and fatty acids. In particular, the phytochemical profile and chemotype can vary depending on climate conditions, soil characteristics, and cultivar differences, which are closely linked to fluctuations in pharmacological activities such as antioxidant, antibacterial, and wound healing effects. This variability in components due to environmental and genetic factors is a crucial consideration when interpreting the functional and therapeutic potential of JA. Typically, the plant reaches a height of 300–450 cm, with aerial parts including flowers, leaves, and stems [21]. The underground portion consists of roots, stolons, and fleshy tubers, which vary in shape—round, pear-shaped, or oval—and resemble potatoes [22]. The color of the tubers ranges from pale brown to dark brown, red, purple, or white, depending on the local climate and environmental conditions [6]. This form of JA is as shown in Figure 2.

## 3. Traditional Medicinal Uses of JA

This review applied a conceptual framework that categorizes evidence into two groups based on mechanism of action to interpret the health effects of JA more clearly. The first category comprises prebiotic-based evidence centered on inulin, encompassing metabolic effects such as gut microbiota regulation, blood glucose control, and improved lipid metabolism. The effects in this category are primarily explained by indirect mechanisms via the gut–metabolic axis, and relatively consistent evidence has been reported in human studies and nutritional intervention studies.

On the other hand, the second category is based on evidence from bioactive components other than inulin, including polyphenols, flavonoids, phenolic acids, sesquiterpenes, and minerals. These components exhibit more direct cellular and molecular-level actions, such as antioxidant, anti-inflammatory, antibacterial, skin-protective, and antihypertensive effects, and their mechanisms have primarily been demonstrated through in vitro and animal studies. The effects in this category may be expressed through pathways that are independent of or complementary to inulin.

This review conceptually separates the functional effects of Jerusalem artichoke into inulin-centered metabolic regulation and pharmacological actions mediated by non-inulin bioactive components. This conceptual distinction is useful for understanding traditionally reported efficacy in relation to modern pharmacological mechanisms. To clarify this further, a summary table aligning traditional uses with their corresponding pharmacological rationale is provided. By doing so, it aims to structure evidence previously presented in a mixed manner across existing studies and more clearly demonstrate the independent and complementary contributions of each component group.

JA is a versatile plant valued for its nutritional, medicinal, and industrial applications. Its edible tubers can be eaten raw or cooked and are rich in carbohydrates, proteins, fiber, and inulin, making them beneficial for both humans and livestock [6,17,23]. Inulin acts as a prebiotic, supporting digestive and immune health, and is widely used as a low-calorie sugar and fat substitute in functional foods, particularly for individuals with diabetes and obesity [24,25,26,27]. Previously reported digestive improvements, anti-inflammatory effects, and wound healing benefits can be interpreted as partially associated with the antioxidant activity, gut microbiota regulation, and enzyme inhibitory activity of inulin and polyphenols, as demonstrated in recent studies. However, a significant portion of the efficacy attributed to folk medicine is based on empirical observations, necessitating additional systematic pharmacological verification of individual traditional claims.

Traditionally, the plant has been used in folk medicine to treat pain, wounds, digestive issues, arthritis, rheumatism, and skin disorders. To more intuitively correlate these traditional uses with the pharmacologic evidence reported to date, this study presents a table summarizing both folk applications and modern experimental and clinical research findings. The folk medicinal use of JA varies by region. In Europe and Russia, it was primarily consumed raw or boiled to alleviate digestive disorders and joint pain. In East Asia, it has been used in dried or sliced form to treat skin conditions and reduce inflammation. Traditional uses based on regional application methods and formulations have been reported in diverse ways, and the corresponding modern pharmacological evidence for each application is summarized in the table.

Meanwhile, apart from these folk medicinal and dietary uses, JA also holds significant value in industrial applications that focus on the physical and chemical properties of its components rather than their physiological activity. Industrially, inulin extracted from JA is converted into fermentable sugars for biofuel and organic acid production, as well as biodegradable polymers [6,28,29]. Furthermore, JA can be consumed raw, boiled, pickled, or stewed, with its flavor varying by preparation—from a strong, nutty taste when raw to a mild, sweet celery-like flavor when boiled. Pickled JA promotes the growth of beneficial lactic acid bacteria and the production of antimicrobial metabolites due to its high inulin content, which acts as a prebiotic [30]. However, brewing JA into “JA tea” can lead to high acrylamide levels, making it unsafe to consume; soaking JA in 1% acetic acid at 20 °C for 1 h can help prevent this. Since acrylamide intake is linked to increased cancer risk, proper preparation is crucial [31]. Moreover, the nutritional and functional properties of JA—such as antioxidant capacity and enzyme-inhibitory activities—vary depending on the processing and drying methods used. Among these, freeze-dried JA retains the highest polyphenolic content (up to 2.7 times more than cooked JA), with 25 identified hydroxycinnamic phenolic acids totaling 820 mg/100 g. Thus, JA serves as a valuable crop for nutrition, health, and sustainable bioenergy development [32].

## 4. Bioactive Compounds from JA

The study identified several chemical components in JA, including flavonoids, phenolic acids, and polysaccharides, which were recognized as the primary active compounds, along with other minor constituents Table 1.

The fibroblast-based wound scratch assay is a cost-effective, widely used method for evaluating the wound-healing potential of natural products. An in vitro wound-healing scratch assay was conducted using National Institutes of Health 3T3 mouse fibroblast cell line (NIH 3T3). Cells were treated with different concentrations of the JA ethyl acetate fraction (Ht-EAF; 25, 50, and 100 µg/mL) and incubated for 48 h. Cell migration was observed at 0, 12, 24, and 48 h, and wound closure was calculated [33,34,35,36]. Compared with the untreated control, *Helianthus tuberosus* ethyl acetate fraction (Ht-EAF) significantly enhanced cell migration in a time- and dose-dependent manner, with the 100 µg/mL concentration showing the greatest effect on wound closure [37,38]. Future studies should elucidate how JA participates in key wound healing mechanisms beyond fibroblast migration, including angiogenesis, collagen deposition, and inflammation regulation. These results suggest JA as a valuable natural ingredient for wound-healing and skin-repair applications. The experimental results are summarized in Table 2. And the detailed characteristics of the included studies are summarized in Supplementary Table S1.

## 5. Pharmacological Activities

**Figure 3:**
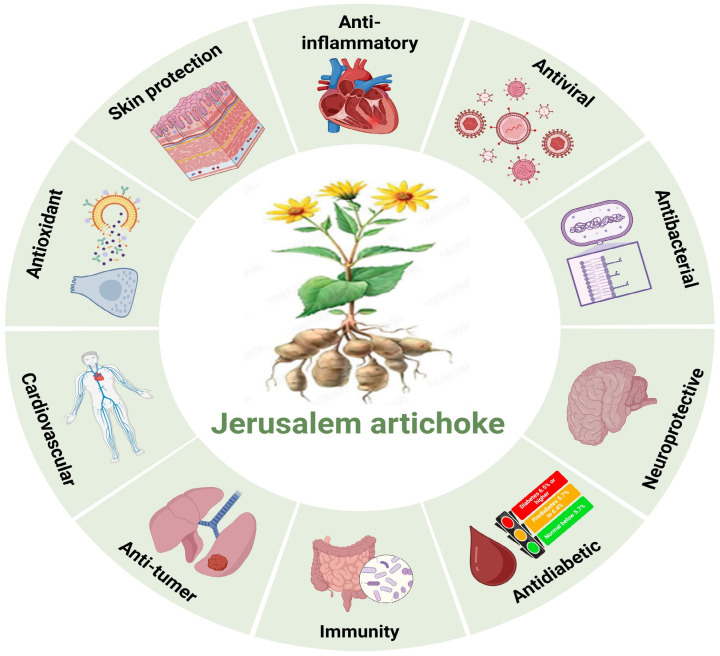
Comprehensive molecular mechanism of JA and its bioactive derivatives-mediated pharmacological actions.

JA and its bioactive derivatives exhibit a wide range of pharmacological and health-promoting effects by modulating various cellular signaling pathways. These pathways are associated with oxidative stress, antioxidant defense, inflammation, cardiovascular protection, cancer suppression, glucose metabolism, and antimicrobial and antiviral activities, highlighting JA’s multifunctional therapeutic potential across diverse health conditions Figure 3.

### 5.1. Anti-Oxidant Properties of JA

The JA leaf extract effectively scavenged 2,2-diphenyl-1-picrylhydrazyl (DPPH), 2,2-azino-bis(3-ethylbenzothiazoline)-6-sulfonic acid (ABTS), and hydroxyl radicals. This result can be interpreted as suggesting the potential for a protective role in pathological processes associated with oxidative stress [43]. A similar study also reported that the methanol extract of JA flowers exhibited antioxidant and α-glucosidase inhibitory activities, attributed to its phenolic compounds, including chlorogenic acid derivatives, flavonoids, and phenols [46]. Distinct effects of JA leaf and tuber extracts on reactive oxygen species (ROS) generation were observed in HaCaT cells. The extracts increased superoxide dismutase (SOD)-1 expression in fibroblast cell lines, whereas Nox-4 expression remained unchanged. Interestingly, the opposite effect was observed in keratinocytes. This demonstrates that the antioxidant response of JA extracts may vary depending on cell type, suggesting that caution is needed when interpreting effects in actual physiological environments [47]. Free radicals are unstable molecules that contain one or more unpaired electrons and readily react with and oxidize biological macromolecules, leading to cellular damage. Oxidative stress plays a major role in cell injury and death, contributing to the development of cancer, neurodegenerative disorders, and cardiovascular diseases [48,49]. Antioxidants counteract these effects by donating electrons or hydrogen atoms, thereby stabilizing free radicals. Their solubility and metal-chelating properties help prevent or neutralize oxidative reactions, while their ability to regulate key metabolic enzymes and modulate gene transcription further enhances their protective effects [50]. These antioxidant mechanisms are presented within a general biological context and should be understood as describing potential modes of action rather than as direct evidence proving the physiological effects of JA-derived components.

The flavonoids extracted from JA leaves exhibited stronger antioxidant activity than butylated hydroxytoluene (BHT). Another study examined eight JA cultivars over 60 days of cold storage to assess changes in inulin content, antioxidant capacity, polyphenol content, and enzyme activity. Inulin levels ranged from 582.43 to 809.70 g/kg, with LZJ047 maintaining the highest content. Antioxidant capacity, ferric reducing antioxidant power (FRAP), and DPPH decreased in all cultivars, correlating with inulin loss, while polyphenol content and enzyme activities, peroxidase (POD), catalase (CAT), and SOD varied. Cluster analysis revealed strong correlations between inulin and antioxidant parameters [51]. Chemical antioxidant assays (DPPH, ABTS, FRAP, etc.) are useful for evaluating free radical scavenging capacity, but they have limitations in directly explaining antioxidant effects in vivo. Some animal studies have simultaneously observed improved blood glucose control indicators and insulin sensitivity alongside increased SOD and catalase activity following JA extract intake, suggesting that antioxidant enzyme regulation may be associated with alleviating metabolic stress. Furthermore, reduced ROS production and regulation of antioxidant enzyme expression in skin cells and inflammation models have been reported to be associated with alleviated inflammatory responses and improved skin barrier function, suggesting that JA-derived antioxidant markers may hold functional significance under specific pathological conditions. Therefore, the antioxidant effects of JA should be interpreted based on biologically robust biomarkers, such as ROS regulation and the activity of endogenous antioxidant enzymes like SOD and catalase.

Overall, existing studies consistently report that JA may exhibit antioxidant activity, primarily associated with its content of phenolic compounds, flavonoids, and inulin. These bioactive components effectively scavenge free radicals, reduce oxidative stress, and protect cells from damage. Both tuber and leaf extracts demonstrate significant antioxidant capacity in vitro and in vivo. These results suggest that JA’s antioxidant properties may be associated with changes in specific disease-related biomarkers, supporting the need for further research based on disease-specific models.

### 5.2. Anti-Inflammatory Properties of JA

Inflammation is a physiological defense response to infection or tissue damage, but when it becomes chronic, it contributes to the pathophysiology of various chronic diseases such as cancer, metabolic disorders, and arthritis. Therefore, exploring compounds with anti-inflammatory potential is essential. JA exhibits notable anti-inflammatory activity, helping to prevent and reduce inflammation in the body [52]. The anti-inflammatory effects of JA have been reported not only at the level of the whole extract’s complex action but also at the level of individual bioactive components and polysaccharides isolated from leaves and tubers. This effect is largely attributed to its rich content of phenolic compounds, including caffeic acid, caffeoylquinic acid, and quercetin-7-O-glucoside. Additionally, the high inulin content in JA supports the growth of beneficial gut bacteria, enhances immune function, and helps alleviate inflammation associated with immune deficiency [53].

JA-derived sesquiterpene lactones and low-molecular-weight compounds have been reported to inhibit the NF-κB and MAPK signaling pathways in LPS-induced inflammatory models and reduce the expression of key inflammatory cytokines such as TNF-α, IL-6, IL-1β, and PGE2. These effects were consistently observed not only in RAW 264.7 macrophage and 3T3-L1 adipocyte–macrophage co-culture models but also in animal inflammation models such as xylene-induced ear edema and carrageenan-induced paw edema [54].

JA polysaccharides have been reported to contribute to maintaining immune homeostasis by simultaneously suppressing representative inflammatory cytokines such as TNF-α, IL-1β, and IL-6, while increasing the expression of anti-inflammatory cytokines like IL-10 [55]. Overall, JA and its bioactive compounds exhibit notable anti-inflammatory effects, suppress pro-inflammatory cytokines, inhibit macrophage activation, and reduce oxidative stress-induced inflammation. Both in vitro and in vivo studies support JA’s potential to alleviate chronic inflammation, contributing to its therapeutic value in metabolic, gastrointestinal, and immune-related disorders. However, these anti-inflammatory effects have primarily been confirmed at the cellular and animal study level to date, and additional clinical research is required for human application.

### 5.3. Anti-Cancer Properties of JA

Tumors are complex diseases with high mortality rates and are difficult to treat due to their heterogeneity and rapid progression. Although current anti-tumor therapies, particularly molecularly targeted drugs, have shown success in treating certain malignancies [56], their efficacy is often limited by drug resistance and toxicity to normal tissues. Consequently, the development of safe and effective anti-tumor agents with minimal side effects remains a major research focus. Traditional natural medicines have shown promising potential as sources of novel anti-tumor compounds [57]. Numerous studies have demonstrated that bioactive compounds derived from natural plants can inhibit tumor growth and metastasis, induce cell cycle arrest, and promote apoptosis in cancer cells through multiple molecular mechanisms [58]. However, these anticancer effects are primarily based on preclinical and exploratory results from cell and animal studies and do not directly imply clinical efficacy.

The anticancer effect of this system can be interpreted as a result of JA extract contributing synergistically to the anticancer activity of selenium nanoparticles, rather than the polysaccharides derived from JA themselves. The anti-tumor activity of JA tubers was demonstrated through the development of a nanostructured biomaterial, Jerusalem artichoke polysaccharide (JAP)-selenium nanoparticles (SeNP), produced by reacting sodium selenite with ascorbic acid and capping the resulting nanoparticles with JA tuber extract. JAP-SeNP exhibited strong anti-proliferative effects on mouse mammary carcinoma cells, achieving an inhibition rate of 41.5% and an apoptosis rate of 38.9%, indicating that JA-derived polysaccharides enhance the anti-tumor potential of selenium nanoparticles [59]. Another study evaluated the anti-cancer potential of JA tuber and shell extracts against various breast cancer cell lines. Using the 2,3-bis(2-methoxy-4-nitro-5-sulfophenyl)-2H-tetrazolium-5-carboxanilide assay (XTT assay), both extracts showed dose- and time-dependent cytotoxicity toward tumor cells while being non-toxic to normal breast cells. The extracts also significantly inhibited adhesion and invasion of Michigan Cancer Foundation-7 human breast adenocarcinoma cell line (MCF-7) cells, indicating strong anti-metastatic activity. Tetramethylrhodamine ethyl ester (TMRE) and Annexin V/7-aminoactinomycin D (7AAD) staining confirmed apoptosis via mitochondrial disruption, indicating that both tuber and shell extracts of JA possess potent anticancer activity [60]. In human colorectal adenocarcinoma cell line (HT-29) colon cancer cells, JA extracts suppressed cell proliferation in a dose-dependent manner, achieving up to 78% inhibition at 250 μg/mL. Annexin V assays revealed DNA fragmentation and late apoptosis, while cell cycle analysis showed G1 phase arrest, confirming apoptosis-mediated growth inhibition. Additionally, Jerusalem artichoke extract (JAE) demonstrated antibacterial activity and inhibited lipase and α-amylase, suggesting its potential in both cancer therapy and metabolic regulation [61]. These results suggest that JA extracts are involved in mechanisms related to cancer cell growth inhibition and apoptosis; however, they remain limited for clinically evaluating the potency or selectivity of their effects. Abdel-Hamid et al. [62] evaluated the hepatoprotective effects of JA tubers as an adjuvant to interferon and ribavirin in carbon tetrachloride (CCl_4_)-induced liver fibrosis. Rats treated with the triple combination exhibited hepatic architecture nearly normal compared with CCl_4_-treated controls. JA tubers supplementation enhanced the therapeutic response, restoring normal histological and biochemical parameters while significantly reducing hepatic expression of tumor protein p53, bcl-2-associated x protein (BAX), and transforming growth factor beta (TGF-β). These findings suggest that JA tubers effectively potentiate the anti-fibrotic action of interferon and ribavirin. These results suggest that JA and its bioactive components may exhibit biological activities related to inhibiting cancer cell proliferation, reducing adhesion and invasion, and inducing apoptosis via the mitochondrial pathway. However, these anticancer-related effects are primarily based on preclinical-level evidence observed in cell and animal models and do not directly imply clinical efficacy or selectivity. Therefore, JA-derived components require further investigation through additional studies, including mechanism elucidation, pharmacokinetic properties, and safety evaluation, to assess their potential as adjunctive anticancer candidates. To date, there remains a lack of systematic analysis regarding the pharmacokinetics—specifically absorption, distribution, metabolism, and excretion—and safety margin for JA-derived anticancer candidate compounds. This deficiency must be addressed in future research.

### 5.4. Anti-Diabetic Properties of JA

The global prevalence of diabetes is rising rapidly, posing a major health and economic burden on individuals, families, healthcare systems, and national economies. This growing challenge underscores the urgent need for affordable and effective antidiabetic therapies. Therefore, exploring the therapeutic potential of JA and developing new diabetes treatments based on its bioactive components are of significant importance for improving healthcare outcomes.

Yang et al. [63] investigated whether JA consumption altered insulin sensitivity, insulin secretion capacity, and pancreatic beta cell (β-cell) survival in type 2 diabetic animals. After 8 weeks, the JA group showed reduced visceral fat and significantly improved glucose tolerance compared with diabetic controls. JA consumption boosted insulin secretion, while JA improved liver insulin sensitivity through activation of insulin signaling, insulin receptor substrate 2 (IRS2)—protein kinase B (Akt)—and activated protein kinase (AMPK) pathways, along with lowered phosphoenolpyruvate carboxykinase (PEPCK) expression and reduced β-cell apoptosis. This suggests that JA enhanced insulin resistance and β-cell function in diabetic rats. In addition, JA inulin supplementation significantly reduced diabetic indicators, including blood glucose, hemoglobin A1c (HbA1c), triglycerides, total cholesterol, low-density lipoprotein cholesterol (LDL cholesterol), and serum pro-inflammatory cytokines in high-fat diet and streptozotocin-induced diabetic mice. It also modulated hepatic lipid metabolism by regulating genes involved in lipid and cholesterol synthesis and degradation. Furthermore, JA inulin enriched beneficial gut microbes such as *Prevotellaceae unclassified genus cluster (UCG)-001*, *Parasutterella*, and *Dubosiella*, and altered 89 metabolites linked to amino acid, lipid, vitamin, and nucleotide metabolism [64]. A similar study also revealed that JA inulin treatment reduced food and water intake, body and liver weight, and levels of triglycerides, total cholesterol, high-density lipoprotein cholesterol (HDL cholesterol), and fasting blood glucose in high-fat diet and streptozotocin-induced hyperglycaemic mice. It also modulated hepatic gene expression, reducing the number of differentially expressed genes from 84 in untreated to 22 in treated mice. Moreover, inulin improved intestinal microbial diversity and significantly increased *Bacteroides* abundance [65]. Furthermore, the *Lactobacillus plantarum*-fermented purple JA extract exhibited the strongest α-glucosidase inhibitory activity in vitro. In vivo, treated mice showed significantly lower blood glucose levels compared to controls, along with increased serum insulin and HDL cholesterol levels. Conversely, triglycerides, non-esterified fatty acids, and total cholesterol were significantly reduced after seven weeks of treatment. Additionally, intestinal α-glucosidase activity was partially inhibited in leptin receptor-deficient diabetic mice (db/db mice) [66]. A synthesis of these preclinical studies indicates that JA or JA inulin commonly exhibits antidiabetic effects through improving insulin sensitivity, regulating blood glucose levels, normalizing lipid metabolism, and modulating gut microbiota.

Another study found that consuming a mixture of JA and fermented soybean powder for 12 weeks effectively reduced postprandial glucose and oxidative stress, particularly 8-epi-prostaglandin F2 alpha (8-epi-PGF2α), in individuals with impaired fasting glucose, impaired glucose tolerance, or newly diagnosed type 2 diabetes [67]. However, this study is a dietary intervention study based on a relatively short period and a limited number of participants, so caution is needed in interpreting the results to generalize the effects of JA alone. In a study using Wistar rats fed a high-fructose diet, JA supplementation (10%) improved fructose-induced insulin resistance and hepatic triglyceride accumulation. Transcriptome analysis revealed that fructose altered the expression of genes involved in fatty acid synthesis malic enzyme 1 (Me1), fibrosis decorin (Dcn), and inflammation cytochrome P450 family 1 subfamily A member 2 (Cyp1a2), nicotinamide phosphoribosyltransferase (NAMPT), whereas JA restored their expression, suggesting that dietary JA may help prevent type 2 diabetes and non-alcoholic fatty liver disease [68]. The crosstalk between macrophages and adipocytes exacerbates adipose tissue inflammation, contributing to insulin resistance and type 2 diabetes. A novel compound from JA, methyl 2-(4′-methoxy-4′-oxobutanamide) benzoate (compound 1), was investigated for its anti-inflammatory potential. Compound 1 reduced lipopolysaccharide-induced secretion of interleukin-1 beta (IL-1β), interleukin-6 (IL-6), and TNF-α in murine macrophage cell line RAW 264.7 (RAW 264.7) macrophages and suppressed pro-inflammatory gene expression (TNF-α, IL-6, IL-1β), monocyte chemoattractant protein-1 (MCP-1), regulated upon activation, normal t-cell expressed and secreted (RANTES), and mitogen-activated protein kinase (MAPK) pathway activation in mouse 3T3-L1 preadipocyte cell line (3T3-L1) adipocytes exposed to macrophage-conditioned media. These results indicate that JA-derived compound 1 may mitigate adipose tissue inflammation and improve insulin sensitivity [54]. Therefore, overall findings show that JA exhibits strong antidiabetic potential. A significant portion of the reported antidiabetic effects is associated with inulin’s regulation of gut microbiota and blood glucose-lowering action, while some effects can be attributed to the anti-inflammatory and insulin signaling-modulating actions of JA-derived specific compounds. Its high inulin content helps regulate blood glucose by improving insulin sensitivity and reducing postprandial glucose levels. JA supplementation also lowers plasma cholesterol and triglycerides while enhancing beneficial gut microbiota, contributing to better metabolic balance. These effects suggest that JA may serve as a natural dietary aid for managing diabetes and related metabolic disorders. Nevertheless, additional long-term human studies comparing the independent effects of JA and inulin are necessary to confirm clinical efficacy.

### 5.5. Neuroprotective Properties of JA

The neuroprotective effects of JA are mainly attributed to its strong antioxidant and anti-inflammatory properties. The plant contains a diverse range of bioactive compounds that help counteract oxidative stress and neuroinflammation, two major contributors to neurodegenerative diseases such as Alzheimer’s disease (AD). Additionally, JA provides proteins that play a crucial role in maintaining neuronal health and managing neurodegenerative conditions [42,69]. The ethanol extracts of JA exhibit neuroprotective potential by inhibiting β-secretase activity, thereby preventing amyloid beta (Aβ) plaque aggregation and preserving cognitive function. JA has also been shown to inhibit cholinesterase activity, thereby enhancing cholinergic transmission in the brain. Furthermore, JA reduces Aβ aggregation and tau phosphorylation through modulation of the glycogen synthase kinase-3 (GSK-3) signaling pathway, as demonstrated in a late-onset *Drosophila* model of AD. These findings suggest that JA possesses promising neuroprotective properties and may serve as a potential therapeutic agent for managing AD pathogenesis [70]. These results suggest the potential neuroprotective effects of JA but should currently be interpreted as hypothesis-generating findings based on limited and heterogeneous preclinical evidence. Another study evaluated the long-term effects of combined treatment with JA, inulin, and fluoxetine on neural stem cell proliferation and cognitive function in AD mice. However, the study design involved the combined use of JA, inulin, and antidepressants, limiting the ability to directly attribute the observed effects solely to JA’s neuroprotective action. Ten-week supplementation with JA, inulin, and fluoxetine improved spatial learning, cognitive memory, and neurogenesis, indicating their safety for brain function. These findings reported that combined treatment with JA, inulin, and fluoxetine shows potential as natural prebiotics that support brain and cognitive function [71]. In particular, it remains unclear whether JA-derived bioactive components can cross the blood–brain barrier, to what extent the dosage used in experiments correlates with human intake levels, and what the key active component mediating the actual neuroprotective effect is. This section condenses detailed descriptions of individual mechanisms and models and reorganizes key findings into summary paragraphs to avoid overemphasizing the limited preclinical evidence for neuroprotective effects. Furthermore, expressions suggesting therapeutic potential should be tempered to a hypothetical level, while the limitations of the evidence and the need for future research should be presented more clearly. Future research should systematically conduct preclinical and clinical studies encompassing the identification of JA-derived neuroprotective active substances, their blood–brain barrier permeability, optimal dose determination, and long-term safety.

### 5.6. Hepatoprotective Effects of JA

The liver performs vital biological functions, including hormone secretion, vitamin and glycogen storage, and the metabolism of carbohydrates, lipids, and proteins. It also plays a crucial role in detoxifying xenobiotics and drugs. However, anti-tuberculosis medications such as ethambutol, isoniazid, and rifampicin can cause hepatotoxicity, leading to liver dysfunction. This damage is typically reflected by elevated serum levels of aspartate aminotransferase (AST), alanine aminotransferase (ALT), alkaline phosphatase (ALP), bilirubin, malondialdehyde (MDA), urea, and creatinine, along with reduced albumin. Notably, natural plants and their phytocompounds have shown potential in protecting the liver by reducing necrosis, oxidative stress, and inflammation, while restoring antioxidant defenses and overall hepatic function. A recent study evaluated the hepatoprotective effects of JA tubers in combination with interferon and ribavirin in CCl_4_-induced hepatotoxic rats. After 8 weeks, rats treated with the triple combination showed nearly normal liver architecture compared to the CCl_4_-treated group. The combination therapy significantly reduced the elevated expression of p53, BAX, and TGF-β observed in CCl_4_-intoxicated rats. These findings suggest that JA tubers enhance the anti-fibrotic and hepatoprotective efficacy of interferon and ribavirin by improving histopathological and biochemical liver functions [62]. These results suggest that JA may serve as a natural therapeutic or dietary supplement to support liver health and prevent hepatic disorders.

### 5.7. Antimicrobial and Antiviral Properties of JA

The antiviral activity of JA was demonstrated through its inhibitory effects on respiratory syncytial virus (RSV) replication, which primarily affects infants and the elderly. A study evaluated the anti-RSV potential of JA polysaccharide, an inulin-type polysaccharide extracted from JA. In vitro, JA polysaccharide showed strong antiviral effects comparable to the standard drug ribavirin, with a half maximal inhibitory concentration (IC_50_) of 29.15 ± 0.44 μg/mL and a selectivity index (SI) of 68.27, while ribavirin showed an IC_50_ of 30.19 ± 0.23 μg/mL and an SI of 76.21. In vivo, JA polysaccharide treatment dose-dependently reduced weight loss, alleviated lung injury, and suppressed RSV replication by lowering viral messenger ribonucleic acid (mRNA) levels in infected mice, suggesting its potential as an effective antiviral agent against RSV [72]. Similarly, JA polysaccharide, an inulin-type β(1→2)-linked D-fructose polymer, was evaluated for its anti-respiratory syncytial virus (RSV) activity in vitro and in vivo. JA polysaccharide exhibited significant antiviral effects with an IC_50_ of about 29.15 μg/mL and effectively reduced RSV proliferation and lung damage in infected mice. It also downregulated toll-like receptor (TLR)3 and TLR4 expression, increased IL-4, and decreased TNF-α and TNF-β levels. These findings suggest that JAP exerts anti-RSV activity through immune modulation and may serve as a promising candidate for RSV therapy [73]. The recent study results demonstrated strong antimicrobial activity of JA compounds against various pathogens. Among the different isolated compounds, ent-kaur-16-en-19-oic acid and β-sitostenone showed notable activity against *Enterococcus faecium*, with MIC values of 6.25–12.50 μg/mL. Additionally, β-sitostenone exhibited anti-tuberculosis activity against *Mycobacterium tuberculosis* with an MIC of 25.00 μg/mL, indicating its potential as a candidate for treating bacterial and Tuberculosis (TB) infections [74]. The antifungal activity of JA extracts was demonstrated against *Rhizoctonia solani*, *Alternaria solani*, and *Botrytis cinerea*. Additionally, extracts obtained with a water–carbon dioxide mixture showed significant antibacterial and antifungal activity against *Staphylococcus aureus*, *Escherichia coli*, *Candida albicans*, and *Candida glabrata*. The CO_2_ + H_2_O extract exhibited minimum inhibitory concentration (MIC) values of 0.62–5 mg/mL for bacteria and 5–10 mg/mL for yeasts, with the strongest activity observed against *Staphylococcus aureus* American-type culture collection (ATCC) 29213 (MIC = 2.5 mg/mL) [75]. Overall, JA and its bioactive compounds exhibit significant antimicrobial and antiviral activities, inhibit the growth of pathogenic bacteria, and promote beneficial microbes such as *Lactobacillus* and *Bifidobacterium*. JA extracts have also demonstrated antiviral potential by suppressing viral replication and enhancing immune defense mechanisms. These properties highlight JA as a promising natural agent for maintaining microbial balance and supporting immune health.

### 5.8. Wound Healing Properties of JA

Cutaneous wound healing is a complex process involving inflammation, proliferation, re-epithelialization, and remodeling to restore skin integrity after injury. While most surgical wounds heal naturally, some become chronic due to infection or dehiscence, with pressure ulcers alone causing around 60,000 deaths and 2 million hospitalizations annually [76,77]. Current research focuses on developing more effective treatments that reduce costs and promote scar-free healing. Conventional therapies often result in scarring, whereas regenerative approaches aim to restore full skin function. Medicinal plants offer promising, cost-effective alternatives with unique antibacterial mechanisms and greater safety compared to synthetic drugs [78].

The study evaluated the wound-healing effects of an ozonated JA tuber ointment in a rabbit full-thickness skin wound model. However, this effect may result from the combined action of both the JA component itself and the antibacterial and tissue regeneration effects of ozone treatment, and a direct comparison with a JA-only formulation has not been conducted.

The ointment, prepared from JA tubers and ozonated using a Herrmann generator, showed strong antibacterial activity in agar diffusion assays. Thirty rabbits were divided into control and treatment groups; the latter received topical ozonated JA tuber ointment twice daily for 5 days. Compared with controls, treated wounds showed significantly greater contraction, enhanced re-epithelialization, and improved histological healing on Days 7, 14, and 21, suggesting that ozonated JA ointment possesses potent tissue-regenerative properties and effectively promotes wound healing [79]. Furthermore, as the results are based on a relatively limited number of animals and a short observation period, additional verification is required regarding long-term therapeutic effects and reproducibility. This study did not include direct comparisons with commercially available standard wound treatments (e.g., antibiotic ointments, silver-based preparations), limiting its ability to assess clinical superiority.

The agar well diffusion assay confirmed JA’s antibacterial activity. High-performance liquid chromatography (HPLC) analysis revealed that its powder primarily contained three major fatty acids as active components. Moreover, the treated group showed a significantly (*p* ≤ 0.05) better wound contraction rate and improved histopathological healing compared with the control group [80].

### 5.9. The Modulatory Effects of JA on Intestinal Microbiota

The mammalian gut hosts nearly 40 trillion bacteria collectively known as the microbiota. An imbalance between beneficial and harmful bacteria, known as dysbiosis, has been linked to the onset of many diseases. Increasing evidence suggests that the gut microbiota plays a key role in disease development and is a promising therapeutic target for natural products.

A recent study investigated the protective effects of JA inulin against type 2 diabetes mellitus (T2DM) induced by a high-fat diet and streptozotocin in mice. JA inulin supplementation significantly improved T2DM-related physiological changes by modulating genes involved in inflammation. Gut microbiota analysis showed enrichment of beneficial bacteria, including *Prevotellaceae* and *Dubosiella*, while metabolomic profiling revealed JA inulin regulation of amino acid, lipid, vitamin, and nucleotide metabolism, suggesting that inulin effectively alleviated hepatic lipid abnormalities and gut dysbiosis associated with T2DM [64]. Male pigs were fed diets containing different levels of dried JA for 1 week prior to slaughter to assess its effects on skatole accumulation, gut microflora, and short-chain fatty acids (SCFAs). Increasing JA levels significantly reduced skatole concentrations in the colon and feces and tended to lower those in adipose tissue. JA supplementation also decreased pH and dry matter content in the colon and feces, reduced *Clostridium perfringens* and enterobacteria populations, and increased total SCFA—particularly acetic and valerianic acids. This study suggests that dietary JA effectively lowered skatole levels in a dose-dependent manner, likely due to enhanced SCFA production and modulation of gut microbiota [81]. Another study evaluated the effects of inulin supplementation on gut microbiota and metabolic pathways in children with obesity. Among 143 participants, inulin intake significantly increased microbial diversity and enriched beneficial and butyrate-producing bacteria, including *Bifidobacterium*, *Blautia*, *Megasphaera*, and *Eubacterium coprostanoligenes*. It also modulated key metabolic pathways related to proteasome and riboflavin metabolism, which were closely linked to improved clinical and metabolic outcomes [82]. Valdovska et al. [83] evaluated the effects of JA and probiotics on intestinal defense in weaning pigs. Piglets fed a diet supplemented with both JA (5%) and *Lactobacillus reuteri* and *Pediococcus pentosaceus* showed significantly lower levels of *Enterobacteriaceae* and coliforms, and higher *Lactobacillus* counts compared to controls. Histological analysis revealed improved intestinal morphology and moderate enterocyte regeneration in the combined treatment group. Moreover, expression of β-defensins 2 and 3 increased, indicating enhanced intestinal defense. Therefore, combining JA with probiotics improved gut microbiota balance, immune activity, and intestinal health in weaning pigs. Another study also revealed that both the water-soluble and organic extracts of JA possess bioactive properties that influence SCFA production and gut microbiota composition. Moreover, powdered JA may be more effective than purified inulin supplements in supporting gut health [84]. Moreover, Szewczyk et al. [85] evaluated the long-term effects of JA, inulin, and fluoxetine on cognitive function and gut microbiota in AD mice. Ten-week combined supplementation with JA, inulin, and fluoxetine promoted beneficial gut bacteria without impairing learning, memory, or neurogenesis, indicating their safety for brain function. Overall, combined supplementation with JA, inulin, and fluoxetine show potential as natural prebiotics that support gut microbiota diversity and may benefit the brain–gut–microbiota axis. An experiment was conducted to examine the effects of adding 2% JA to the meat-on-bone diet of captive Indian leopards. JA supplementation did not affect feed intake or nutrient utilization but lowered serum urea and triglyceride levels. It significantly increased beneficial gut bacteria (*Lactobacillus* and *Bifidobacterium*) and short-chain fatty acids (acetate, butyrate, lactate), while reducing fecal ammonia, Clostridia, and pH, indicating that JA improved hindgut fermentation and microbial balance, suggesting it is a beneficial prebiotic supplement for captive leopards [86]. In summary, JA demonstrates potential as a natural prebiotic material that improves gut microbial balance and short-chain fatty acid production in animal models. However, this effect can be interpreted as being largely attributable to the contribution of general prebiotic action mediated by inulin rather than JA’s own inherent components. Furthermore, since most evidence is based on animal studies, careful interpretation is required for direct application to the human gut microbiome. Future research requires systematic verification of the intake dosage applicable to humans, dietary feasibility, and clinical efficacy.

## 6. Acute and Subacute Toxicity

No relevant in-depth toxicity records have been found through research of all relevant literature in databases such as PubMed, ScienceDirect, and Elsevier. However, a recent study evaluated the anticancer effects of its tuber and shell extracts on various breast cancer cell lines. The extracts showed dose- and time-dependent cytotoxicity toward tumor cells without affecting healthy breast cells, demonstrating that both the tuber and shell extracts of JA exhibit no toxicity, and suggesting potential as natural candidates for cancer treatment [60]. Another study investigated the nutritional composition, chemical contaminants, and natural toxins in JA tubers grown in four provinces of Thailand. The tubers contained high levels of fructans and dietary fiber (15.4 ± 0.2 g and 3.2 ± 0.8 g/100 g fresh weight) and were rich in potassium and iron. Contaminant levels—including insecticide residues, heavy metals, nitrates, nitrites, cyanide, and trypsin inhibitors—were very low, indicating the product’s safety and confirming that JA tubers are nutritious and safe for consumption [87]. Currently reported toxicity studies are primarily limited to acute or short-term exposure assessments at the cellular level, and systematic animal testing data on subacute and chronic toxicity remain insufficient. In addition, the immobilized inulinase enzymes from JA showed no mutagenic or cytotoxic effects, indicating their safety for biotechnological applications. Both native and immobilized forms were non-toxic, supporting their potential use in safe enzymatic production of fructose from plant oligosaccharides [88]. To date, no significant toxicity has been observed in studies involving JA extracts. In particular, standardized assessments according to OECD toxicity testing guidelines, the establishment of a no-observed-adverse-effect level (NOAEL), and dose–response relationship analysis have not yet been reported, limiting the ability to definitively establish safety based solely on the current evidence. However, comprehensive toxicity evaluations remain limited. Future research should include systematic in vitro and in vivo animal studies to clarify the safety profile of JA bioactive compounds, determine optimal dosage levels, and assess potential long-term effects. Such studies will be essential for confirming its safety, elucidating mechanisms of action, and supporting its development as a functional food ingredient or therapeutic agent.

## 7. Industrial Application of JA

### 7.1. Food Benefits from JA

JA tubers are widely used in dairy production to enhance the nutritional quality of dairy products. Incorporating JA polysaccharides enables the creation of fat-free or low-fat yogurt enriched with inulin, protein, fiber, vitamins (B1, B2, C), and essential minerals, including potassium, phosphorus, calcium, and magnesium. Studies have shown that substituting sugar with JA syrup or adding dried JA polysaccharides (3–5%) in yogurt and milk desserts improves nutritional and biological value, enhances antioxidant capacity, and helps lower cholesterol and triglyceride levels. Additionally, milk drinks containing JA juice (25–35%) and JA polysaccharides offer preventive health benefits, improved sensory qualities, and greater product diversity at reduced cost [89,90]. Furthermore, beverages incorporating 15–20% mashed JA have been developed by blending it with fruits and vegetables such as carrot, pumpkin, apple, pineapple, and passion fruit. These drinks are rich sources of dietary fiber, with 200 mL providing about 35% of daily fiber needs. The formulations aimed to create low-calorie, well-balanced beverages in which the mild flavor of JA was complemented by stronger fruit flavors. Additionally, JA–soybean fermented beverages were developed, offering good texture and appearance, and regular consumption may support immune function. JA juice formulated for elderly individuals also showed high acceptability, particularly for its color, sweetness, and flavor [89,91]. JA exhibits relatively mild flavor and texture-improving effects, resulting in high sensory acceptability. However, its addition ratio can affect texture, sweetness, and storage stability, necessitating product-specific optimization. In addition, bread and pastry products enriched with JA powder show improved nutritional and functional properties. A 75:25 wheat flour–JA powder blend produced bread with balanced nutrients, low glycemic index, and 226 kcal/100 g. Adding 10–15% JA powder to wheat or rye–wheat bread enhanced yeast fermentation, improved texture and volume, and delayed staling, due to its fiber content (inulin and pectin). Similarly, incorporating JA powder into pastries and cookies (10–30%) increased dietary fiber, mineral, and inulin levels, while improving dough quality and reducing preparation time [92,93,94,95]. However, most of the reported health-related effects correspond to functional potential based on product composition and nutritional analysis, and clinical efficacy has been verified only to a limited extent. These findings suggest that JA is a versatile functional ingredient in food production, valued for its high inulin, fiber, and mineral content. Its incorporation into products such as bread, yogurt, and beverages enhances nutritional quality, supports gut health, and lowers the glycemic index, making it suitable for individuals with diabetes and obesity. Furthermore, for the commercial application of JA-based functional foods, regulatory requirements such as country-specific food ingredient approvals, functional claims, and daily intake standards must also be considered. Moreover, it improves texture, stability, and shelf life while reducing calories and fat, making JA and its bioactive ingredients an ideal component for developing health-promoting food formulations. Due to these characteristics, JA is evaluated as a promising functional ingredient that can be utilized in the development of health-oriented foods.

### 7.2. Cosmetic Benefits from JA

The cosmetic industry is one of the fastest-growing sectors worldwide. Plant-based cosmetic ingredients play a key role due to their rich content of biologically active compounds, broad range of effects, safety, and easy accessibility. As a result, they offer numerous benefits and versatile applications. Plant-based raw materials have complex chemical compositions and can be used in products for consumers of all ages and skin types. They are also suitable for both primary and supportive treatments of various dermatological conditions.

A study found that JA extracts—both aqueous and aqueous-ethanolic—are effective for skin protection, have antioxidant properties, and work well in body wash formulations. Inulin, a JA-derived compound, proved safe and reduced skin irritation by about 40% across concentrations. There was also no difference in inulin between the two extract types. Overall, both JA extracts and inulin show promise as multifunctional cosmetic ingredients with added prebiotic benefits that support healthy skin flora and protect the skin and mucous membranes [96]. Another study evaluated the effect of JA tuber extract on the properties of a two-phase makeup remover. The ultrasound-extracted extract showed strong antioxidant activity and promoted fibroblast proliferation without cytotoxicity. Incorporating the extract (0–15%) did not affect product stability, and patch tests confirmed skin safety. These findings suggest that JA extract is a valuable cosmetic ingredient with antioxidant and skin-friendly properties suitable for formulation in makeup removers [97]. Inulin, extracted from JA, is used to investigate cosmetic, moisturizing, and skin-protective properties. Results show that it helps retain skin hydration, supports a healthy microbiome, and reduces redness and irritation. It may influence indicators related to anti-aging through antioxidant and moisturizing mechanisms. Inulin also shows antimicrobial activity, making it useful as a natural preservative. Modified forms, such as acetylated inulin, enhance stability and delivery of active ingredients. Owing to its natural origin and multifunctional benefits, inulin is a valuable ingredient in modern skincare and personal care formulations [98]. It is functionally similar to existing moisturizing ingredients such as hyaluronic acid and glycerin. Therefore, these findings indicate that JA is a promising natural ingredient for cosmetic applications due to its abundance of bioactive compounds, such as inulin, polyphenols, flavonoids, and vitamins. Its extracts exhibit strong antioxidant, moisturizing, and skin-regenerating properties, helping protect against oxidative stress and the signs of aging. Inulin also acts as a prebiotic, promoting healthy skin microbiome and hydration. With proven safety, stability, and antimicrobial properties in formulations, JA offers great potential for developing effective, eco-friendly skincare and cosmetic products.

## 8. Conclusions

Research findings on the physiological effects of JA show considerable variation between studies, which can be explained by several factors. First, the content of inulin, polyphenols, and flavonoids varies depending on the cultivar, which may result in differences in the magnitude and direction of antioxidant and metabolic regulation effects. Furthermore, the stability and bioavailability of bioactive components may vary depending on the processing and storage conditions of the raw materials, whether heat treatment is applied, and the drying method used.

The solvents used in the extraction process (water, ethanol, methanol, etc.) also directly influence the composition of the extracted compounds, leading to differences in the reported pharmacological effects. Furthermore, many studies lack standardization of active ingredients, limiting the comparison of dosages and the interpretation of results. This heterogeneity and inconsistency across studies suggest caution is needed when generalizing the functionality of Jerusalem artichoke, and future research requires systematic study designs that consider variety characteristics, extraction conditions, and component standardization.

In summary, this paper offers a comprehensive overview of the traditional uses, medicinal properties, phytochemical composition, and pharmacological benefits of JA and its bioactive compounds.

Furthermore, the physiological effects of JA vary depending on the matrix form used (whole extract vs. single isolated compound) and the plant part (leaves vs. tubers). According to existing studies, whole extracts exhibit relatively broad antioxidant, anti-inflammatory, and metabolic regulatory effects due to synergistic interactions among various bioactive components. In contrast, single isolated compounds are advantageous for precise validation of effects related to specific mechanisms of action, but their scope of efficacy may be limited when applied in vivo.

By plant part, tubers are high in inulin and dietary fiber, primarily contributing to metabolic health effects such as regulating gut microbiota balance and improving blood glucose and lipid metabolism. In contrast, leaves are rich in polyphenols and sesquiterpene lactones, reported to be more directly involved in anti-inflammatory, antioxidant, antibacterial, and skin-related physiological activities. These differences suggest the potential for strategic differentiation of Jerusalem artichoke based on its intended use as a functional food, nutraceutical, or material derived from specific functional components.

**Figure 4:**
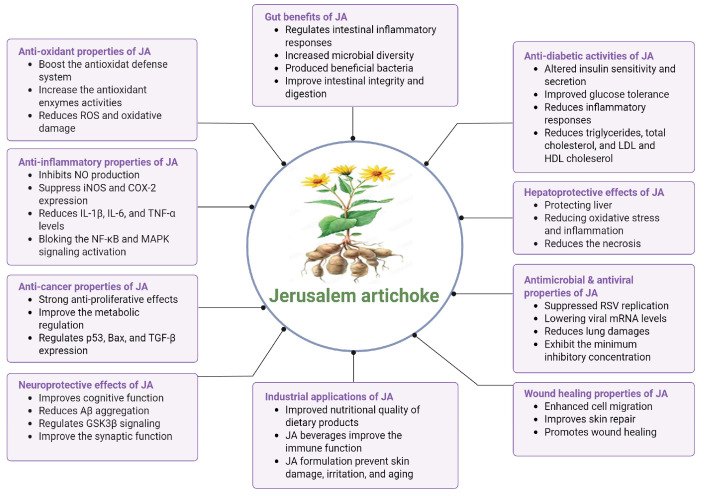
Health benefits of consuming JA and its bioactive derivatives.

As shown in Figure 4, previous studies have reported the potential for JA and its bioactive components to exhibit physiological activities related to antioxidant, anti-inflammatory, and metabolic regulation; however, a significant portion of these effects is based on in vitro and animal experiments. Due to its high inulin and dietary fiber content, physiological responses related to gut microbiota balance and lipid and glucose metabolism have been observed; however, its clinical efficacy for managing obesity or diabetes has not yet been sufficiently established. Some preclinical studies have reported that JA induces biological responses related to skin hydration, reduction in roughness, and wound healing; however, this does not imply clinical efficacy.

Rich in polyphenols and nutrients, JA can be consumed raw, boiled, or as an extract. In summary, JA and its bioactive components have been reported to potentially exhibit physiological activities related to antioxidants, anti-inflammation, and metabolic regulation; however, these effects have primarily been demonstrated in preclinical studies and from a nutritional perspective.

However, a significant portion of the studies included in this review are limited to in vitro and animal experiments, and clinical evidence in humans remains limited. Therefore, future studies should systematically verify the safety, efficacy, and optimal intake of JA through randomized controlled trials with sufficient sample sizes. Furthermore, differences in plant species, extraction solvents, processing conditions, and active ingredient standardization limit the comparability and generalizability of research findings.

However, these future research directions should be interpreted with the understanding that sufficient clinical evidence has not yet been accumulated. However, several key areas require further investigation. First, future research should aim to isolate and identify the individual bioactive components of JA and elucidate their effects on specific molecular targets and physiological indicators related to inflammation, glucose metabolism, and antioxidant activity. Second, in vivo studies incorporating quantitative neurological assessment indicators such as cognitive function improvement, neuroinflammation markers, and oxidative stress indicators should be conducted to verify the neuroprotective effects of JA-derived components. Third, the goal should be to verify the causal relationship between traditional claims and scientific evidence by linking JA’s traditional uses and efficacy to modern pharmacological indicators (such as anti-inflammatory markers and metabolic indicators). Fourth, for industrial and pharmaceutical applications, systematic subacute and acute toxicity studies are required to establish NOAELs and derive safe dosage ranges for each part and extract of JA. Lastly, future in vivo studies should establish as primary endpoints the clarification of the absorption, distribution, metabolism, and excretion characteristics of JA extracts and their major active components, thereby defining their bioavailability and effective concentration range. Overall, this paper highlights both the progress and the gaps in JA research, providing a foundation for future studies aimed at unlocking its full medicinal and functional potential.

JA is highly adaptable to cultivation and yields stable harvests, making it cost-effective for securing raw materials for functional foods and health functional ingredients. In particular, key bioactive components, including inulin, demonstrate excellent industrial scalability as they can be mass-produced through existing food processing methods. However, component stability may decrease during processing and storage, and standardization and quality control of extracts and isolated components remain key challenges for industrial applications. Furthermore, to commercialize JA-derived materials, in particular, for the commercialization of JA-derived materials, securing clinical safety and functional evidence, including human application trials that comply with each country’s food and health functional food regulatory systems, is essential.

## Figures and Tables

**Table 1:** Traditional uses of JA and corresponding modern pharmacological evidence.

Traditional Use (Ethnomedicine)	Plant Part/Preparation	Major Bioactive Components	Type of Evidence	Corresponding Modern Pharmacological Effects
Digestive disorders, gut discomfort	Tuber (raw, boiled, pickled)	Inulin, dietary fiber	Human, in vivo	Prebiotic effects, modulation of gut microbiota, improved digestion and lipid metabolism
Diabetes and blood glucose control	Tuber (dietary intake, extract)	Inulin	Human, in vivo	Regulation of blood glucose levels, improved insulin sensitivity, metabolic regulation
Obesity and metabolic imbalance	Tuber (whole extract)	Inulin, polysaccharides	Human, in vivo	Reduced lipid accumulation, improved lipid profiles, appetite and weight management
Anti-inflammatory use (general)	Leaves, tuber (extract)	Polyphenols, flavonoids, phenolic acids	In vitro, in vivo	Anti-inflammatory activity via modulation of inflammatory mediators and oxidative stress
Wound healing and skin disorders	Tuber (topical extract)	Polyphenols, sesquiterpene lactones	In vitro, in vivo	Enhanced wound healing, skin barrier protection, anti-inflammatory and antioxidant effects
Joint pain, arthritis, rheumatism	Leaves, tuber (decoction)	Polyphenols, sesquiterpenes	In vitro, in vivo	Antioxidant and anti-inflammatory effects potentially related to pain and inflammation relief
Antioxidant and anti-aging purposes	Leaves, tuber (extract)	Polyphenols, flavonoids	In vitro, in vivo	Free radical scavenging, cellular protection against oxidative damage
Antibacterial and antifungal use	Leaves (extract)	Polyphenols, sesquiterpenes	In vitro	Antibacterial and antifungal activity against selected pathogens

**Table 2:** Evidence-ranked bioactive compounds identified in JA.

Evidence Level	Compound	Plant Part	Experimental Model	Key Endpoints	Reported Activity	Ref.
Human	Inulin-rich JA	Tuber	Older adults	Postprandial glucose, gut microbiota	Morning intake reduced postprandial glucose and improved gut microbiota	[6]
In vitro	Phenolic-rich extract	Tuber, Leaf	HaCaT, BJ fibroblasts	Antioxidant activity, cytotoxicity	High antioxidant activity with low cytotoxicity	[33]
In vitro	Kaurenoic acid	Flowers	Bacterial/immune cells	Inflammatory markers, growth inhibition	Antibacterial, anti-inflammatory	[34]
In vitro	Heliangin	Aerial part	LPS-induced RAW 264.7 cells	NO, inflammatory mediators	Anti-inflammatory effect via NF-κB inhibition	[35]
In vitro	Ethyl acetate fraction	Tuber	HepG2, NIH 3T3 cells	Glucose uptake, wound closure	Improved glucose metabolism and enhanced wound healing	[38]
In vitro	Chlorogenic acid	Tuber	Inflammatory cell models	Cytokines, ROS	Anti-inflammatory, antioxidant	[39,40]
In vitro	Ferulic acid	Tuber	Cancer/metabolic cells	Oxidative stress, glucose metabolism	Antioxidant, antidiabetic	[41]
In vitro	Butein	Leaves	Cancer cells	Apoptosis	Anticancer	[42]
In vitro	Kaempferol 3-O-glucoside	Leaves	Microbial/oxidative assays	Growth inhibition, ROS	Antioxidant, antimicrobial	[42,43,44]
In vitro	Heliangin	Leaves	Vascular/immune cells	Inflammatory signaling	Anti-inflammatory, anti-atherosclerotic	[45]
In vitro	Vanillin	Whole plant	Cell-based assays	Glucose metabolism, inflammation	Antidiabetic, anti-inflammatory	[42]
In vitro	4,15-isoatriplicolide	Whole plant	Cancer cell lines	Cell proliferation	Anticancer	[46]
In vitro & in vivo	Inulin	Inulin-Se nanoparticles	Cancer cells + mice	Tumor growth	Drug delivery enhancement	[47]

## Data Availability

No new data were created or analyzed in this study.

## Supplementary Materials

The following supporting information can be downloaded at: https://www.mdpi.com/article/10.3390/cimb48020180/s1.

## Abbreviations

WHO—world health organization; JA—Jerusalem artichoke; NOS—Newcastle Ottawa scale; BHT—hydroxytoluene; DPPH—2,2-diphenyl-1-picrylhydrazyl; ABTS—2,2-azino-bis(3-ethylbenzothiazoline)-6-sykfibuc acid; ROS—reactive oxygen species; SOD—superoxide dismutase; FRAP—ferric reducing antioxidant power; POD—peroxidase; CAT—catalase; TNF-α—tumer necrosis factor-α; LPS—lipopolysaccharide; ICAM—intercellular adhesion molecule; VCAM—vascular cell adhesion molecule; MCP—monocyte chemoattractant protein; NF-κB—nuclear factor kappa B; IκBα—inhibitor of kappa B alpha; JAP—Jerusalem artichoke polysaccharide; SeNP—selenium nanoparticles; XTT assay—2,3-bis(2-methoxy-4-nitro-5-sulfophenyl)-2H-tetrazolium-5-carboxanilide assay; MCF-7—Michigan cancer foundation-7 human breast adenocarcinoma cell line; TMRE—tetramethylrhodamine ethyl ester; 7AAD—7-aminoactinomycin D; HT-29—human colorectal adenocarcinoma cell line; JAE—Jerusalem artichoke extract; CCl_4_—carbon tetrachloride; p53—tumor protein p53; BAX—bcl-2-associated X protein; TGF-β—transforming growth factor beta; β-cell—pancreatic beta cell; IRS2—insulin receptor substrate 2; Akt—protein kinase B; AMPK—activated protein kinase; PEPCK—phosphoenolpyruvate carboxykinase; HbA1c—hemoglobin A1c; LDL (cholesterol)—low-density lipoprotein cholesterol; HDL (cholesterol)—high-density lipoprotein cholesterol; db/db mice—leptin receptor-deficient diabetic mice; IL-1β—interleukin-1 beta; IL-6—interleukin-6; MCP-1—monocyte chemoattractant protein-1; RANTES—regulated upon activation, normal t-cell expressed and secreted; 8-epi-PGF2α—8-epi-prostaglandin F2 alpha; RAW 264.7—murine macrophage cell line RAW 264.7; 3T3-L1—mouse 3T3-L1 preadipocyte cell line; MAPK—mitogen-activated protein kinase; Me1—malic enzyme 1; Dcn—decorin; Cyp1a2—cytochrome P450 family 1 subfamily A member 2; Nampt—nicotinamide phosphoribosyltransferase; UCG—unclassified genus cluster; AD—Alzheimer’s disease; Aβ—amyloid beta; GSK-3—glycogen synthase kinase-3; AST—aspartate aminotransferase; ALT—alanine aminotransferase; ALP—alkaline phosphatase; MDA—malondialdehyde; RSV—respiratory syncytial virus; IC_50_—half maximal inhibitory concentration; SI—selectivity index; mRNA—messenger ribonucleic acid; TLR—Toll-Like Receptor; MIC—minimum inhibitory concentration; TB—Tuberculosis; ATCC—American Type Culture Collection; HPLC—high-performance liquid chromatography; NIH 3T3—National Institutes of Health 3T3 mouse fibroblast cell line; Ht-EAF—*Helianthus tuberosus* ethyl acetate fraction; T2DM—type 2 diabetes mellitus; SCFAs—short-chain fatty acids; NOAEL—no-observed-adverse effect level.
